# The differential effects of heat-shocking on the viability of spores from *Bacillus anthracis*, *Bacillus subtilis*, and *Clostridium sporogenes* after treatment with peracetic acid- and glutaraldehyde-based disinfectants

**DOI:** 10.1002/mbo3.277

**Published:** 2015-07-17

**Authors:** Jordon K March, Michael D Pratt, Chinn-Woan Lowe, Marissa N Cohen, Benjamin A Satterfield, Bruce Schaalje, Kim L O'Neill, Richard A Robison

**Affiliations:** 1 Department of Microbiology and Molecular Biology, 4007-B LSB, Brigham Young UniversityProvo, Utah, 84602; 2 Department of Statistics, 230 TMCB, Brigham Young UniversityProvo, Utah, 84602

**Keywords:** Anthrax, *Bacillus anthracis*, disinfection, heat-shock, inactivation kinetics, spore, sporicide

## Abstract

This study investigated (1) the susceptibility of *Bacillus anthracis* (Ames strain), *Bacillus subtilis* (ATCC 19659), and *Clostridium sporogenes* (ATCC 3584) spores to commercially available peracetic acid (PAA)- and glutaraldehyde (GA)-based disinfectants, (2) the effects that heat-shocking spores after treatment with these disinfectants has on spore recovery, and (3) the timing of heat-shocking after disinfectant treatment that promotes the optimal recovery of spores deposited on carriers. Suspension tests were used to obtain inactivation kinetics for the disinfectants against three spore types. The effects of heat-shocking spores after disinfectant treatment were also determined. Generalized linear mixed models were used to estimate 6-log reduction times for each spore type, disinfectant, and heat treatment combination. Reduction times were compared statistically using the delta method. Carrier tests were performed according to AOAC Official Method 966.04 and a modified version that employed immediate heat-shocking after disinfectant treatment. Carrier test results were analyzed using Fisher's exact test. PAA-based disinfectants had significantly shorter 6-log reduction times than the GA-based disinfectant. Heat-shocking *B. anthracis* spores after PAA treatment resulted in significantly shorter 6-log reduction times. Conversely, heat-shocking *B. subtilis* spores after PAA treatment resulted in significantly longer 6-log reduction times. Significant interactions were also observed between spore type, disinfectant, and heat treatment combinations. Immediately heat-shocking spore carriers after disinfectant treatment produced greater spore recovery. Sporicidal activities of disinfectants were not consistent across spore species. The effects of heat-shocking spores after disinfectant treatment were dependent on both disinfectant and spore species. Caution must be used when extrapolating sporicidal data of disinfectants from one spore species to another. Heat-shocking provides a more accurate picture of spore survival for only some disinfectant/spore combinations. Collaborative studies should be conducted to further examine a revision of AOAC Official Method 966.04 relative to heat-shocking.

## Introduction

The decontamination efforts that followed the intentional release of *Bacillus anthracis* spores through the US Postal Service have generated significant interest in chemical disinfectants that are capable of inactivating spores from virulent strains of *B. anthracis*. In addition, sporicidal disinfectants are important in a variety of clinical settings. For example, dental instruments, surgical instruments, and endoscopes require treatment between uses that ensures sufficient spore inactivation to prevent cross-contamination (Angelillo et al. 1998; Rutala et al. 1998; Rutala and Weber 2004). Alkaline glutaraldehyde and peracetic acid are common high-level disinfectants that might be used after a biological attack or in a healthcare setting; both are commercially available and highly effective sporicides (Russell 1990; Spotts Whitney et al. 2003).

Glutaraldehyde is an effective sporicide that displays optimal antimicrobial activity under basic conditions (Hopwood et al. 1970; Power and Russell 1990; Russell 1990; Coates 1996; Angelillo et al. 1998; Tennen et al. 2000). Consequently, aqueous glutaraldehyde solutions are activated by the addition of alkalinizing agents. Glutaraldehyde inactivates spores by cross-linking outer proteins and blocking normal germination events (Power and Russell 1989; Tennen et al. 2000). CIDEX™ (Advanced Sterilization Products, Irvine, CA) is a 2.4% alkaline glutaraldehyde solution that has been available commercially for many years (Lane et al. 1966). CIDEX™ was investigated throughout this study because it is a frequently used high-level disinfectant and cold sterilant.

Peracetic acid is an effective disinfectant against a wide-variety of microorganisms such as viruses, fungi, vegetative bacterial cells, and bacterial endospores (Kline and Hull 1960; Hussaini and Ruby 1976; Baldry 1983; Leaper 1984; Marquis et al. 1995; Setlow et al. 2002). In fact, it has been shown to be an effective disinfectant against spores from *B. anthracis* (Hussaini and Ruby 1976). Its primary mechanism of action involves the generation of hydroxyl and organic radicals (Clapp et al. 1994; Marquis et al. 1995). These radicals lead to the oxidation of the double bonds found in carbohydrates, nucleic acids, lipids, and proteins (Marquis et al. 1995). STERIPLEX™ (SBIOMED LLC, Orem, UT) formulations contain peracetic acid and other proprietary active ingredients, which create a synergistic antimicrobial effect when combined. Preliminary studies in our laboratory indicated that STERIPLEX™ solutions have rapid sporicidal activity. In addition, oral toxicity studies in rats, conducted in other laboratories, demonstrated that these solutions are minimally cytotoxic and have relatively few corrosive properties (data not shown). STERIPLEX™ HC (0.25% peracetic acid) and STERIPLEX™ Ultra (1.3% peracetic acid) were the formulations used throughout this study.

Upon exposure to chemical disinfectants, bacterial endospores can be unaffected, sublethally injured, or irreparably damaged (i.e., inactivated or killed). Upon neutralization of the disinfectant and incubation in or on nutrient media, sublethally injured spores can recover and convert back to vegetative growth through the process of germination. The spores that fail to germinate are generally considered to be dead. However, treating spores with lysozyme or sodium hydroxide after disinfectant treatment can promote the germination of spores thought to be irreparably damaged (Gorman et al. 1983; Dancer et al. 1989). In addition, exposing spores to high heat (heat-shocking) after treatment with certain biocides has been shown to aid in the revival of injured spores (Spicher and Peters 1976, 1981; Gorman et al. 1983; Williams and Russell 1993).

Although the treatments described above are not typically practiced in clinical settings, the ability to revive spores that were supposedly killed by exposure to a chemical disinfectant should not be ignored. Experiments involving these harsh treatments have revealed the risk of overestimating the effectiveness of certain disinfectants against various spore species (Gorman et al. 1983; Power et al. 1989; Russell 1990; Williams and Russell 1993). In fact, the official method for determining the activity of sporicidal disinfectants, as published by AOAC International, requires spores to be heat-shocked at 80°C for 20 min after disinfectant treatment and incubation, to avoid overestimating the effectiveness of a given chemical disinfectant (AOAC Official Method 966.04^2006^). However, the results of heat-shocking spores after peracetic acid treatment have not been investigated. In addition, previous studies on the effects of heat-shocking on spore viability have not included spores from virulent *B. anthracis* strains.

In this study, suspension tests were conducted, with and without heat-shocking, to obtain the inactivation kinetics for CIDEX™, STERIPLEX™ HC, and STERIPLEX™ Ultra against spores from a fully virulent strain of *B. anthracis* as well as spores from well-characterized strains of *Bacillus subtilis* and *Clostridium sporogenes*. These tests provided comparative data on the efficacy of the three disinfectants against the different spore species. They also allowed the investigation of the effects of heat-shocking (according to AOAC Official Method 966.04) on the viability of spores in suspension after treatment with glutaraldehyde and peracetic acid. In addition, these tests revealed the consequences of heat-shocking on virulent strains of *B. anthracis* after disinfectant treatment.

The disinfectants were also tested against spores from *B. subtilis* and *C. sporogenes* that were deposited on carriers. This was done according to AOAC Official Method 966.04 and a modified version that employed immediate heat-shocking after disinfectant treatment. This allowed for the evaluation of the effects of a 3-week incubation period prior to heat-shocking that is prescribed by AOAC Official Method 966.04.

## Materials and Methods

### Bacterial strains

Bacterial strains used in this study were *B. anthracis* A0462 (the virulent Ames strain), *B*. *subtilis* ATCC 19659, and *C. sporogenes* ATCC 3584. The identity of *B. anthracis* was confirmed by the gas chromatographic analysis of cellular fatty acids using an Agilent 6890 Series Gas Chromatograph (Santa Clara, CA) and software purchased from MIDI (Newark, DE). Real-time PCR assays targeting unique chromosomal and plasmid gene sequences (Tetracore Inc, Rockville, MD) were used to definitively identity the strain as *B. anthracis* and to confirm the presence of both virulence plasmids. Vegetative cultures of *B. anthracis* and *B. subtilis* were grown on Columbia agar (Becton, Dickinson and Company, Sparks, MD) and incubated under aerobic conditions, whereas vegetative cultures of *C. sporogenes* were grown on Reinforced Clostridial Agar (RCA, Becton, Dickinson and Company) and incubated under anaerobic conditions using an Anoxomat system (Advanced Instruments, Inc., Norwood, MA).

### Laboratory conditions

Procedures involving *B. anthracis* were performed under Biosafety Level 3 (BSL-3) operating conditions. All other procedures were conducted under Biosafety Level 2 (BSL-2) operating conditions.

### Disinfectants

The following common high-level disinfectants were tested against spores from *B. anthracis*, *B. subtilis*, and *C. sporogenes*: CIDEX™ Activated Dialdehyde Solution, STERIPLEX™ HC, and STERIPLEX™ Ultra. Disinfectants were tested as recommended by the manufacturer. Additionally, all disinfectants were activated immediately before testing.

### Neutralizing agents

Controls were conducted to ensure adequate neutralization of the disinfectants by combining 100 *μ*L of a spore suspension (containing approximately 1 × 10^4^ spores mL^−1^) with 1 mL of disinfectant and 9 mL of the appropriate neutralizer. A 1% (w/v) glycine solution, prepared just prior to use, was used to neutralize the aldehyde-based disinfectant. The peracetic acid-based disinfectants were inactivated using a freshly prepared neutralizing solution which was formulated as follows: 500 mmol L^−1^ Tris pH 8.0, 12.72% Tween 80, 6% Tamol SN, 1.7% lecithin, 1.1% catalase, 1% cysteine, and 1% peptone. The neutralized solution (containing approximately 100 spores mL^−1^) was allowed to rest for 20 min before being assayed for the number of viable spores. Neutralizer controls were plated in triplicate using a membrane filtration system (E-Z Pak 0.45 *μ*m, Millipore Corporation, Billerica, MA).

### Spore suspensions

Aliquots of a saturated bacterial culture (100 *μ*L) were used to inoculate flasks containing 250 mL of Leighton-Doi Broth (Leighton and Doi 1971). The flasks were incubated at 32°C with vigorous shaking for approximately 3–4 days, or until the culture exhibited >95% refractile spores. The percent of refractile spores was monitored on a daily basis by phase-contrast microscopy. The culture was transferred to 50 mL conical vials and heated for 30 min at 65°C to kill vegetative cells. The spores were pelleted by centrifugation at 5000*g* for 15 min at 4°C and suspended in 20 mL ice-cold sterile HPLC water. After incubating for 16–18 h at 4°C to promote the lysis of dead vegetative cells, the spore suspensions were centrifuged, as described above, and washed three times in 20 mL ice-cold sterile HPLC water to further purify the spore suspensions. Spore suspensions of *B. anthracis* and *B. subtilis* were prepared as described above, whereas a spore suspension of *C. sporogenes* was purchased from Presque Isle Cultures (Erie, PA). All spore suspensions were quantified using serial dilution and plated in triplicate using membrane filtration. The spore titers for each spore suspension are listed in Table1. All spore suspensions were stored at 4°C until used.

**Table 1:** Spore suspension concentrations

Organism	Concentration (spore mL^−1^)
*Bacillus anthracis*	1.80 × 10^9^–1.98 × 10^9^
*Bacillus subtilis*	4.16 × 10^9^–7.02 × 10^9^
*Clostridium sporogenes*	1.24 × 10^7^–3.12 × 10^7^

### Suspension tests

A spore suspension was vortexed for 2–3 min to ensure a homogenous mixture. Aliquots of the spore suspension (1 mL) were transferred to vials containing 9 mL of the chosen disinfectant (previously equilibrated to 20°C) at time zero. The vial was vortexed and placed back in a 20°C water bath.

Samples of the spore/disinfectant suspension were taken after various exposure times. The vial was removed from the water bath and vortexed before a 1 mL aliquot of the solution was removed to 9 mL of an appropriate neutralizing agent. The spore/neutralizer suspension was vortexed and allowed to stand for 20 min to allow complete neutralization of the active ingredient. The neutralized solution was serially diluted in physiological saline solution (PSS) and the viable spores of 1 mL aliquots from each dilution were quantified in triplicate using membrane filtration. After samples from each dilution were plated, the dilution tubes were heat-shocked in an 80°C water bath for 20 min. After heat-shocking, the viable spores of 1 mL aliquots from each dilution were again quantified in triplicate using membrane filtration. In the case of *B. anthracis* and *B. subtilis*, filter membranes were incubated on plates containing Columbia agar, whereas prereduced RCA plates were used for *C. sporogenes*. All plates were incubated at 37°C. *B. anthracis* and *B. subtilis* colonies were counted after incubating for 24 and 48 h, whereas *C. sporogenes* colonies were counted only after 48 h in order to ensure that anaerobic conditions were maintained. Assays were repeated in triplicate for each species-disinfectant combination.

### Carriers

Polyester suture loops and porcelain penicylinders were used throughout the investigation. Polyester suture loops were used instead of the typical silk suture loops, because the latter have been shown to interact with peracetic acid (McDonnell 2003). All carriers were prepared by, and purchased from Presque Isle Cultures. Preparation of the carriers was done according to AOAC Official Method 966.04 and included (1) inoculation with spores of *B. subtilis* ATCC 19659 or *C. sporogenes* ATCC 3584, (2) spore enumeration, and (3) verification of spore resistance to hydrochloric acid.

Presque Isle Cultures reported spore titers that exceeded 1 × 10^6^ spores per carrier. As described below, these titers were confirmed prior to use. Five carriers were randomly selected from each lot number. Each carrier was transferred to a different 50 mL conical vial containing 10 mL nutrient broth (Becton, Dickinson and Company) and 2% Tween. The vials were sonicated for 10 min in a Sonicor™ SC-200 ultrasonic cleaner (Sonicor, Wallingford, CT) in order to dislodge spores from the carrier material. Following sonication, the vials were vortexed for 2 min to further remove spores from the carrier. The spore suspensions were serially diluted in PSS and the number of viable spores in each dilution was quantified by membrane filtration of 1 mL samples. Samples from each dilution were plated in triplicate. In the case of *B. subtilis*, filter membranes were incubated on plates containing Columbia agar, whereas prereduced RCA plates were used for *C. sporogenes*. All plates were incubated at 37°C. *B. subtilis* colonies were counted after incubating for 24 and 48 h, whereas *C. sporogenes* colonies were counted after 48 h. Plate counts were used to determine the average spore titer for each lot number. The average spore titers are listed in Table2.

**Table 2:** Concentrations of spores dried onto carriers from recovery experiments

*Bacillus subtilis*		
Lot 032107^1^	Carrier	Average titer (cfu)
	Porcelain penicylinder	1.05 × 10^6^
	Polyester suture loop	1.46 × 10^6^
Lot 052507^2^	Carrier	Average titer (cfu)
	Porcelain penicylinder	1.12 × 10^6^
	Polyester suture loop	1.00 × 10^6^
*Clostridium sporogenes*		
Lot 040207^1^	Carrier	Average titer (cfu)
	Porcelain penicylinder	1.21 × 10^6^
	Polyester suture loop	3.05 × 10^6^
Lot 052607^2^	Carrier	Average titer (cfu)
	Porcelain penicylinder	1.61 × 10^6^
	Polyester suture loop	5.27 × 10^6^

1 Lots used for CIDEX™ and STERIPLEX™ Ultra tests.

2 Lots used for STERIPLEX™ HC.

### Carrier tests

**Figure 1:**
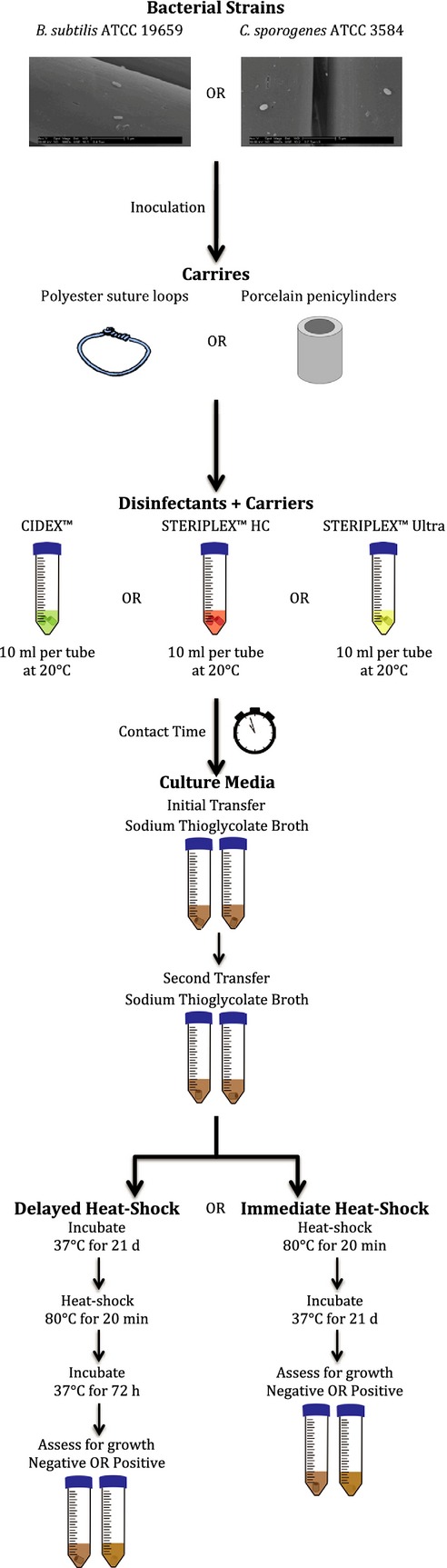
Polyester suture loops and porcelain penicylinders were prepared and tested according to AOAC Official Method 966.04 (See “Delayed Heat-Shock”) and a modified version (See “Immediate Heat-Shock”). Spores from *B. subtilis* had an exposure time of 4 h when tested against CIDEX™, whereas spores from *C. sporogenes* had an exposure time of 1 h when tested against CIDEX™. Regardless of spore species, the exposure times for STERIPLEX™ HC and STERIPLEX™ Ultra were 25 min and 15 min, respectively. Exposure times were determined experimentally by previous testing (data not shown) to be at the end of the kill curve for each spore species-disinfectant combination. Thirty polyester suture loops and 30 porcelain penicylinders were tested, for each spore type, disinfectant, and heat-treatment combination.

Polyester suture loops and porcelain penicylinders were used to evaluate the effectiveness of the disinfectants against spores deposited on solid surfaces. Experiments were performed according to AOAC Official Method 966.04 (Fig.1). Aliquots of a disinfectant (10 mL) were transferred to 50 mL conical vials and placed in a 20°C water bath. After equilibrating in the water bath for 10 min, two polyester suture loops or two porcelain penicylinders were placed in each vial using a flamed metal hook. Once the specified contact time had elapsed, the carriers were removed using a flamed metal hook and placed in separate vials containing Sodium Thioglycolate Broth (STB, Becton, Dickinson and Company). After the initial transfer had been completed, each carrier was again transferred to a second vial of STB. Vials were incubated at 37°C for 21 days. After the initial incubation period, the vials were heat-shocked at 80°C for 20 min and incubated at 37°C for an additional 72 h, after which vials were assessed for growth. Thirty polyester suture loops and 30 porcelain penicylinders were tested, as described above, for each spore species-disinfectant combination.

Spores from *B. subtilis* had an exposure time of 4 h when tested against CIDEX™, whereas spores from *C. sporogenes* had an exposure time of 1 h when tested against CIDEX™. Regardless of spore species, the exposure times for STERIPLEX™ HC and STERIPLEX™ Ultra were 25 min and 15 min, respectively. Exposure times were determined experimentally by previous testing (data not shown) to be at the end of the kill curve for each spore species-disinfectant combination.

A modified version of AOAC Official Method 966.04 was performed with the following alteration: the vials were heat-shocked at 80°C for 20 min immediately following transfer to the second tube of STB instead of incubating for 21 days before being heat-shocked (Fig.1). It is important to note that the vials were not heat-shocked a second time after 21 days. Thirty polyester suture loops and 30 porcelain penicylinders were tested, as described above, for each spore species-disinfectant combination.

### Statistical methods

#### Suspension tests

Suspension tests were used to obtain the inactivation kinetics for the three disinfectants against spores from *B. anthracis*, *B. subtilis*, and *C. sporogenes*, with and without heat-shocking. On each test day, a different combination of spore species and disinfectant was selected at random until each combination was repeated three times. Each dilution assayed for viable spores was plated in triplicate and these counts were averaged to obtain an estimate for each dilution.

A generalized linear-mixed model (GLMM) was fitted to the viable spore count data for each of the three spore species using the GLIMMIX procedure of the SAS® software (SAS Institute Inc, Cary, NC). The GLMMs were specified with the Poisson distribution as the basic distribution, the logarithm as the link function, and the dilution factor of each count as an offset. For each spore species, the logarithm of the expected bacterial count was modeled as a linear or quadratic function of time, with distinct polynomial coefficients for all combinations of disinfectants and heat-shock treatments. For each of the heat-shock treatments, a common intercept was specified for all disinfectants. Samples and triplicate determinations within samples were modeled as random effects in the GLMMs.

Estimated parameters of the GLMMs were combined to estimate 6-log_10_ reduction times along with their standard errors and covariances for each spore species, disinfectant, and heat-shock combination. Six-log_10_ reduction times were compared statistically among spore species, disinfectants, and heat-shock treatments using the delta method.

#### Carrier tests

The number of positive tests after delayed heat-shock was compared to the number of positive tests after immediate heat-shock using the FREQ procedure of the SAS® software. For this data, Fisher's exact test was used.

## Results and Discussion

### Suspension tests

**Figure 2:**
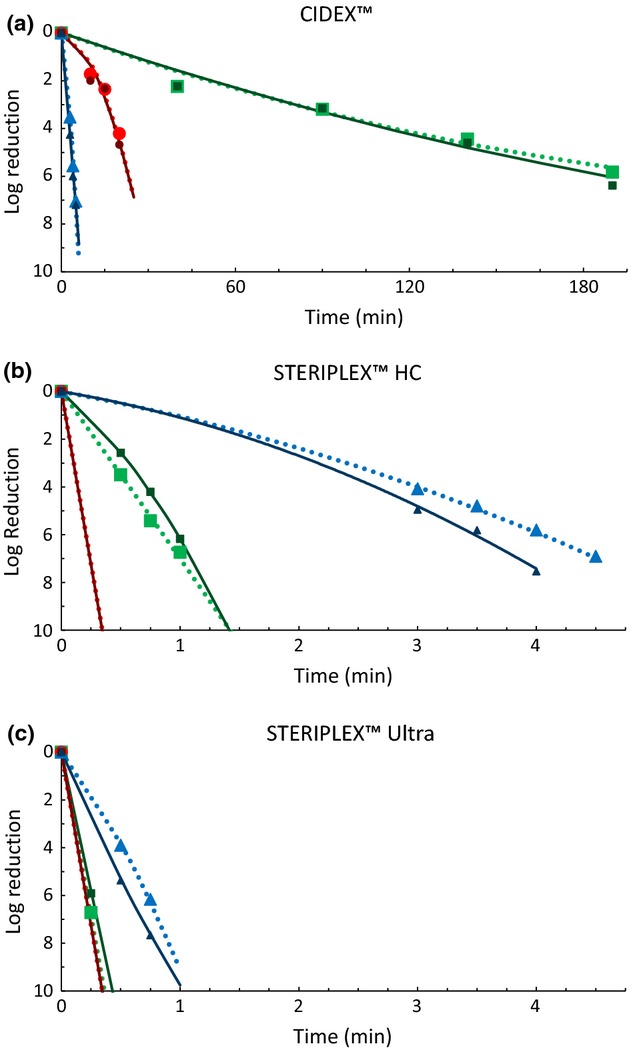
The inactivation kinetics of spores from *Bacillus anthracis* (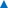 and 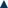), *Bacillus subtilis* (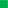 and 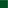), and *Clostridium sporogenes* (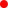 and 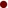) upon treatment with CIDEX™, STERIPLEX™ HC, and STERIPLEX™ Ultra. The bright shapes and dotted lines (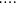) represent the observed values and fitted curves for spores not heat-shocked. The dark shapes and solid lines (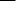) represent the observed values and fitted curves for heat-shocked spores.

**Figure 3:**
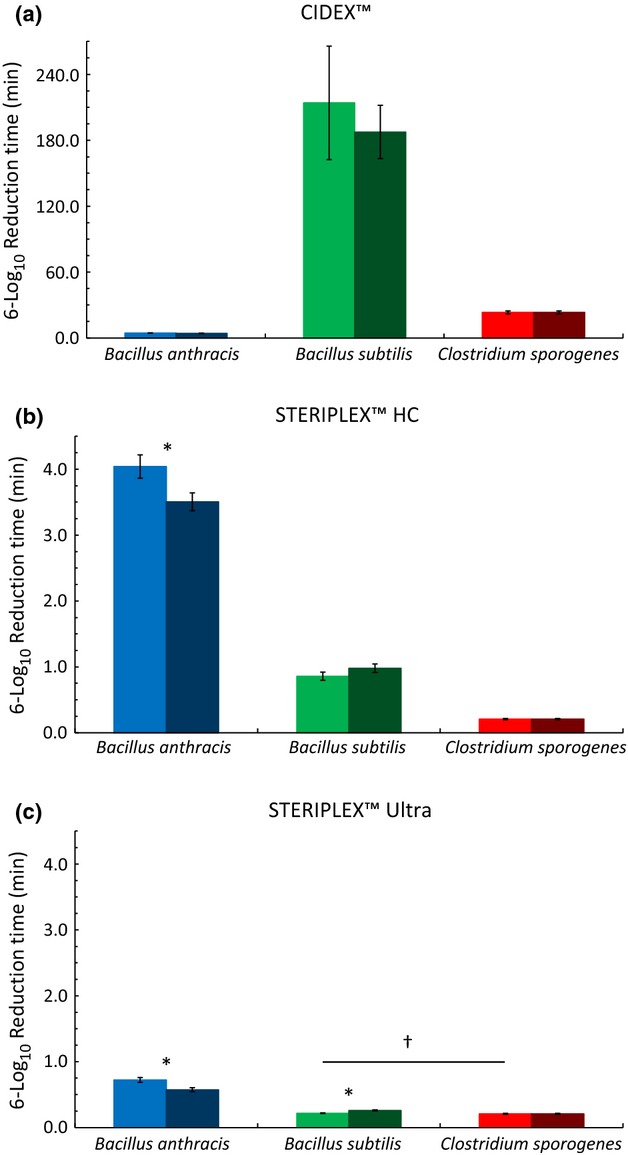
The estimated 6-log_10_ reduction times for spores from *Bacillus anthracis* (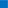 and 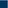), *Bacillus subtilis* (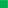 and 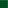), and *Clostridium sporogenes* (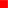 and 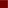) after treatment with CIDEX™, STERIPLEX™ HC, and STERIPLEX™ Ultra. The bright bars represent spores that were not heat-shocked, whereas the dark bars represent spores that were heat-shocked. Significant differences between heat treatments, but within the same spore species are denoted by (*). Differences across spore species that are not significant are denoted by (†).

The first objective of this study was to investigate the effects of heat-shocking (as described in AOAC Official Method 966.04) on the viability of spores from *B. anthracis*, *B. subtilis*, and *C. sporogenes* in suspension after treatment with CIDEX™, STERIPLEX™ HC, and STERIPLEX™ Ultra. This was accomplished in part by comparing the inactivation kinetics for the disinfectants against each of the three spore species, with and without heat-shocking (Fig.2A–C). Additionally, generalized linear-mixed models were used to estimate 6-log_10_ reduction times for each spore type, disinfectant, and heat treatment combination. The 6-log_10_ reduction times and the approximate standard errors for each of the combinations are shown in Table3. A comparison of 6-log_10_ reduction times from each spore species, with and without heat-shocking, within each disinfectant is shown in Figure3A–C.

**Table 3:** Estimated 6-log_10_ reduction times and approximate standard errors for spores from *Bacillus subtilis, Bacillus anthracis,* and *Clostridium sporogenes* using the three different disinfectants

Species	Disinfectant	Heat-shock	6 log_10_ reduction time (min)	
			Estimate	SE
*Bacillus anthracis*	CIDEX™	−	4.37	0.16
		+	4.14	0.19
	STERIPLEX™ HC	−	4.04	0.18
		+	3.50	0.14
	STERIPLEX™ Ultra	−	0.72	0.04
		+	0.57	0.03
*Bacillus subtilis*	CIDEX™	−	214.04	51.65
		+	187.57	24.32
	STERIPLEX™ HC	−	0.86	0.06
		+	0.98	0.07
	STERIPLEX™ Ultra	−	0.22	0.01
		+	0.26	0.01
*Clostridium sporogenes*	CIDEX™	−	23.22	1.40
		+	23.20	1.43
	STERIPLEX™ HC	−	0.21	0.01
		+	0.21	0.01
	STERIPLEX™ Ultra	−	0.21	0.01
		+	0.21	0.01

All three spore species, regardless of heat treatment, differed significantly from each other in 6-log_10_ reduction times when exposed to CIDEX™ (Fig.3A). Additionally, heat-shocking the spore suspensions after treatment with CIDEX™ did not result in a significant change in any of the 6-log_10_ reduction times (Fig.3A). Upon exposure to CIDEX™, spores from *B. anthracis* had the shortest 6-log_10_ reduction times, whereas spores from *B. subtilis* had the longest (Fig.2A and Table3). In fact, spores from *B. subtilis* were approximately 50 times more resistant to CIDEX™ than were the spores from *B. anthracis* (Table3). Spores from *C. sporogenes* had 6-log_10_ reduction times that were significantly different from the other two species, but were more similar to *B. anthracis* than *B. subtilis* (Fig.2A and Table3). Spores from *C. sporogenes* were about five times more resistant to CIDEX™ than the spores from *B. anthracis* (Table3).

Upon exposure to STERIPLEX™ HC, all three spore species, regardless of heat treatment, once again displayed significantly different 6-log_10_ reduction times from each other (Fig.3B). Heat-shocking the spore suspensions after treatment with STERIPLEX™ HC did not result in a significant change in the 6-log_10_ reduction times for spores from *B. subtilis* and *C. sporogenes* (Fig.3B). However, heat-shocking spores from *B. anthracis* after disinfectant treatment caused a significant decrease in the 6-log_10_ reduction times (Fig.3B). When tested against STERIPLEX™ HC, *C. sporogenes* spores had the shortest 6-log_10_ reduction times, whereas spores from *B. anthracis* had the longest reduction times (Fig.2B and Table3). In this case, spores from *B. anthracis* were approximately 20 times more resistant to STERIPLEX™ HC than were the spores from *C. sporogenes* (Table3). Spores from *B. subtilis* had 6-log_10_ reduction times that were significantly different from the other two species, but were more similar to those of *C. sporogenes* than those of *B. anthracis* (Fig.2B and Table3); spores from *B. anthracis* were roughly four times more resistant to STERIPLEX™ HC than were the spores from *B. subtilis* (Table3). It is also important to note that the reduction times for spores from *B. anthracis*, in the absence of heat-shocking, were not significantly different when comparing STERIPLEX™ HC to CIDEX™.

Upon exposure to STERIPLEX™ Ultra, spores from *B. subtilis* and *C. sporogenes* did not differ significantly in their 6-log_10_ reduction times prior to being heat-shocked (Fig.3C). After heat treatment, spores from *C. sporogenes* had significantly shorter reduction times than those from *B. subtilis* (Fig.3C). This was not due to a change in the reduction times of spores from *C. sporogenes*; rather heat-shocking caused a significant increase in the reduction times of spores from *B. subtilis* (Fig.3C). In either situation, with heat-shock or without heat-shock, spores from *B. subtilis* and *C. sporogenes* had significantly shorter reduction times than those from *B. anthracis* (Fig.3C). Although spores from *B. anthracis* had the longest reduction times for either heat condition, they once again showed a significant decrease in 6-log_10_ reduction times after being heat-shocked (Fig.3C). Also, the reduction times for spores from *C. sporogenes*, regardless of heat treatment, were not significantly different when comparing STERIPLEX™ HC to STERIPLEX™ Ultra.

In general, STERIPLEX™ HC and STERIPLEX™ Ultra displayed extremely rapid sporicidal activity (Fig.2A–C). STERIPLEX™ Ultra had the most rapid sporicidal activity across all spore species (Fig.2A–C and Table3), resulting in a 6-log_10_ reduction in as little as 15–45 sec (Fig.3C and Table3). CIDEX™ had comparable activity to STERIPLEX™ HC on spores from *B. anthracis*; however, it was generally much slower than the two peracetic acid-based disinfectants on the other spore species.

These results also showed important differences between the three spore species with respect to sporicide resistance. Spores from *B. subtilis* proved to be less resistant than those from *B. anthracis* to STERIPLEX™ HC and STERIPLEX™ Ultra. Greater exposure times (up to 3.5 times greater) were required for *B. anthracis,* to achieve the level of kill seen in *B. subtilis* (Figs.2B–C and 3B–C). However, the opposite is true of CIDEX™. In this case, *B. subtilis* spores were significantly more resistant than those from *B. anthracis*, requiring a 186 min longer exposure time to achieve a similar inactivation (Figs.2A and 3A).

*C. sporogenes* spores proved to be extremely susceptible to STERIPLEX™ HC and STERIPLEX™ Ultra, but less so to CIDEX™. A complete kill, or a >6-log_10_ reduction was always observed within 15 sec with the two peracetic acid-based disinfectants, whereas a 6-log reduction took approximately 23 min with CIDEX™. It is not surprising that spores from *C. sporogenes*, an anaerobic bacterium, are more susceptible to oxidative damage than those from aerobic bacteria such as *B. subtilis* and *B. anthracis*.

This study also provided important information regarding the effects of heat-shocking after disinfectant treatment on spore recovery. For example, heat-shocking did not significantly increase the 6-log_10_ reduction times of any of the spore species following exposure to CIDEX™, which suggests that heat does little to overcome or augment the damage mediated by glutaraldehyde. On the other hand, when *B. subtilis* spores were heat-shocked immediately following exposure to STERIPLEX™ Ultra, a significant level of spore resuscitation was observed. This result indicates that while peracetic acid causes rapid injury to bacterial spores, heat-shocking can aid in their recovery. Interestingly, the resuscitation observed when *B. subtilis* spores were heat-shocked after exposure to STERIPLEX™ Ultra was not observed in *B. anthracis* (Fig.3). In fact, after treatment with STERIPLEX™ HC or STERIPLEX™ Ultra, heat-shocking *B. anthracis* spores resulted in a significant decrease in spore recovery.

Taken together, these data indicate that spore species differ widely in their susceptibility to disinfectants and their response to heat-shocking following disinfectant treatment. These data showed a significant spore species-disinfectant-heat-shock interaction. Because these interactions are complex and unpredictable, tests with and without heat-shocking should be performed when evaluating the sporicidal properties of a disinfectant.

The differences in spore resistance and recovery may be influenced by many factors including, but not limited to, the presence or absence of additional genes on plasmids, chromosomal-based genetic differences between species, and by interactions between gene products of the plasmids and the chromosome. Further research is needed to more specifically determine the reasons for the highly significant species-disinfectant-heat-shock interactions seen in this study. Testing with a larger number of isolates and a wider range of disinfectants may help define the significant variables involved. In a clinical setting, it would be advisable to increase the disinfectant exposure times past the end-points tested here, to ensure complete kill. For disinfection of *B. anthracis*, however, these peracetic acid-based disinfectants are as effective as the glutaraldehyde-based disinfectant, and, in the case of STERIPLEX™ Ultra, much more so. In addition, the use of data from surrogate organisms to model inactivation kinetics of virulent *B. anthracis* strains may be misleading, and caution should be used when extrapolating sporicidal results from one spore species to another, depending on the disinfectant used.

### Carrier tests

The second aim of this study was to evaluate the benefits of the 3-week incubation period prior to heat-shocking that is prescribed by AOAC Official Method 966.04. This was done according to AOAC Official Method 966.04 and a modified version that employed immediate heat-shocking after disinfectant treatment. Spores from *B. anthracis* were not used in these experiments because this organism is not specified for use in the AOAC Official Method 966.04. The results from the carrier tests are shown in Table4. In AOAC Official Method 966.04, results are based on a combined total of carriers (porcelain penicylinders and suture loops) that yielded growth; the results in Table4 are reported similarly.

**Table 4:** Effect of immediate versus delayed heat-shock on the resuscitation of disinfectant-treated spores dried onto carriers

Species	Disinfectant	Exposure time	Delayed heat-shock	Immediate heat-shock	*P* 1
*Bacillus subtilis*	CIDEX™	4 h	3/60	5/60	0.3585
*Clostridium sporogenes*	CIDEX™	1 h	3/60	4/60	0.5
*Bacillus subtilis*	STERIPLEX™ HC	25 min	3/60	9/60	0.0627
*Clostridium sporogenes*	STERIPLEX™ HC	25 min	1/60	1/60	0.7521
*Bacillus subtilis*	STERIPLEX™ Ultra	15 min	2/60	7/60	0.0815
*Clostridium sporogenes*	STERIPLEX™ Ultra	15 min	2/60	4/60	0.3397

1 Fischer's exact test used for this data.

For CIDEX™, a total of three *B. subtilis* carriers were positive when the test was performed according to the AOAC Official Method 966.04 (heat-shock after 3 weeks of incubation). However, five positive tests were observed when carriers were immediately heat-shocked, a 1.67-fold increase (*P* = 0.3585). Three *C. sporogenes* carriers were positive under AOAC guidelines, with four positives produced when carriers were immediately heat-shocked, a 1.33-fold increase (*P* = 0.5000).

For STERIPLEX™ HC, a total of three *B. subtilis* carriers were positive when the test was performed according to the AOAC Official Method 966.04. However, the group subjected to immediate heat-shock had nine positive carriers, a threefold increase (*P* = 0.0627). For *C. sporogenes*, only one carrier was positive in each group (*P* = 0.7521).

For STERIPLEX™ Ultra, two *B. subtilis* carriers were positive when the test was performed according to the AOAC Official Method 966.04, as opposed to seven after immediate heat-shock, a 3.5-fold increase (*P* = 0.0815). Two *C. sporogenes* carriers were positive under AOAC guidelines, and four were positive when subjected to immediate heat-shock, a twofold difference (*P* = 0.3397).

Taken together, these results indicate that, when testing STERIPLEX™ HC, STERIPLEX™ Ultra, and perhaps other peracetic acid-based disinfectants with *B. subtilis*, immediate heat-shock after disinfection may be a better indicator of the effectiveness of the sporicidal activity of a disinfectant than the current AOAC guidelines. In 2003, the Environmental Protection Agency initiated research to improve efficacy test methods for sporicides (Tomasino 2005). Since then, the AOAC Official Method 966.04 has been reevaluated several times (McDonnell and Russell 1999; Miner et al. 2001, 2004; Tomasino 2005; Tomasino and Hamilton 2006; Tomasino and Samalot-Freire 2007), but none of these evaluations addressed the effect of an immediate heat-shock. The findings of this study may warrant further evaluation of the AOAC Official Method 966.04, with regards to this parameter.
